# Extensive Drug Resistance in Malaria and Tuberculosis

**DOI:** 10.3201/eid1607.091840

**Published:** 2010-07

**Authors:** Chansuda Wongsrichanalai, Jay K. Varma, Jonathan J. Juliano, Michael E. Kimerling, John R. MacArthur

**Affiliations:** Author affiliations: US Agency for International Development, Bangkok, Thailand (C. Wongsrichanalai, J.R. MacArthur);; US Centers for Disease Control and Prevention, Beijing, People’s Republic of China (J.K. Varma);; US Centers for Disease Control and Prevention, Atlanta, Georgia, USA (J.K. Varma, J.R. MacArthur);; University of North Carolina School of Medicine, Chapel Hill, North Carolina, USA (J.J. Juliano);; The Bill and Melinda Gates Foundation, Seattle, Washington, USA (M.E. Kimerling)

**Keywords:** Malaria, antimicrobial resistance, tuberculosis and other mycobacteria, CME, perspective

## Abstract

Emergence of artemisinin resistance is reason to revise the definition of drug resistance for malaria.

## MEDSCAPE CME ACTIVITY

Medscape, LLC is pleased to provide online continuing medical education (CME) for this journal article, allowing clinicians the opportunity to earn CME credit. This activity has been planned and implemented in accordance with the Essential Areas and policies of the Accreditation Council for Continuing Medical Education through the joint sponsorship of Medscape, LLC and Emerging Infectious Diseases. Medscape, LLC is accredited by the ACCME to provide continuing medical education for physicians. Medscape, LLC designates this educational activity for a maximum of 0.5 *AMA PRA Category 1 Credits*™. Physicians should only claim credit commensurate with the extent of their participation in the activity. All other clinicians completing this activity will be issued a certificate of participation. To participate in this journal CME activity: (1) review the learning objectives and author disclosures; (2) study the education content; (3) take the post-test and/or complete the evaluation at http://cme.medscape.com/viewpublication/30063; (4) view/print certificate.

## Learning Objectives

Upon completion of this activity, participants will be able to:

- Define multidrug-resistant tuberculosis and describe effective management strategies.
- Identify multidrug-resistant malaria and discuss management issues.

## EDITOR

**P. Lynne Stockton, VMD, MS**, copy editor, Emerging Infectious Diseases. Disclosure: P. Lynne Stockton, VMD, MS, has disclosed no relevant financial relationships.

## CME AUTHOR

**Charles P. Vega, MD**, Associate Professor; Residency Director, Department of Family Medicine, University of California, Irvine. *Disclosure: Charles P. Vega, MD, has disclosed no relevant financial relationships.*

## AUTHORS

Disclosures: Chansuda Wongsrichanalai, MD, DrPH; Jay K. Varma, MD; Jonathan J. Juliano, MD, MSPH; and John R. MacArthur, MD, MPH, have disclosed no relevant financial relationships. Michael E. Kimerling, MD, MPH, has disclosed that he owns stock in Amgen, Inc.; Johnson & Johnson Pharmaceutical Research & Development, LLC; Merck & Co., Inc.; and Novartis Pharmaceuticals Corporation
