# Supplementary material for: Extensive Drug Resistance in Malaria and Tuberculosis
Source: Emerg Infect Dis. 2010 Jul;16(7):1063–7. doi: 10.3201/eid1607.091840 (PMC3321906; doi:10.3201/eid1607.091840)
Supplement: Related texts

# Review article: Extensive Drug Resistance in Malaria and Tuberculosis

**DOI:** 10.3201/eid1607.091840

**Authors:** Chansuda Wongsrichanalai, Jay K. Varma, Jonathan J. Juliano, Michael E. Kimerling, John R. MacArthur

**Affiliations:** Author affiliations: US Agency for International Development, Bangkok, Thailand (C. Wongsrichanalai, J.R. MacArthur);; US Centers for Disease Control and Prevention, Beijing, People’s Republic of China (J.K. Varma);; US Centers for Disease Control and Prevention, Atlanta, Georgia, USA (J.K. Varma, J.R. MacArthur);; University of North Carolina School of Medicine, Chapel Hill, North Carolina, USA (J.J. Juliano);; The Bill and Melinda Gates Foundation, Seattle, Washington, USA (M.E. Kimerling)

## Abstract

Drug resistance in malaria and in tuberculosis (TB) are major global health problems. Although the terms multidrug-resistant TB and extensively drug-resistant TB are precisely defined, the term multidrug resistance is often loosely used when discussing malaria. Recent declines in the clinical effectiveness of antimalarial drugs, including artemisinin-based combination therapy, have prompted the need to revise the definitions of and/or to recategorize antimalarial drug resistance to include extensively drug-resistant malaria. Applying precise case definitions to different levels of drug resistance in malaria and TB is useful for individual patient care and for public health.

Malaria and tuberculosis (TB) are 2 of the most common infectious diseases in resource-limited countries. Each year, >1.7 million persons die of TB (*1*) and almost 1 million die of malaria (*2*). For each disease, emergence of resistance to common first-line therapies has been a major challenge to disease control. Drug resistance in TB and malaria arises from inadequate or inappropriate use of antimicrobial agents; however, the definitions used to classify drug resistance, as well as the public health control measures, vary. These differences sometimes lead to confusion and misinterpretation by those unfamiliar with either or both diseases. This confusion is compounded by the fact that each pathogen is increasingly resistant to more drugs, and new descriptive terms such as multidrug resistant (MDR) and, for TB, extensively drug-resistant (XDR) have been introduced to describe these changes. For TB, definitions of these new terms are now widely accepted. For malaria, recent changes in clinical effectiveness of antimalarial drugs, in particular the emergence of artemisinin resistance, are forcing malaria experts to consider revising the definitions of drug resistance for *Plasmodium falciparum.* Without clear definitions, prioritization of resources to treat and control malaria will be difficult.

## Categories of Drug Resistance

### Tuberculosis

Drug-resistant TB was identified shortly after the first anti-TB drugs were introduced in the 1940s (*3*); the term refers to TB strains resistant to at least 1 anti-TB drug, usually determined by in vitro phenotypic methods (e.g., mycobacterial culture). Globally, resistance to a single anti-TB drug is the most common pattern of drug resistance. Recognition of the relatively rapid onset of resistance to anti-TB monotherapy, usually within months, led to the development of multidrug therapy as the standard of care in the 1960s (*4*).

Although TB can be treated with many drugs and a strain of TB can be resistant to any or all of these, an international consensus evolved to define MDR TB as resistance to at least isoniazid and rifampin. Resistance to other anti-TB drugs, without resistance to both isoniazid and rifampin, is defined as polydrug resistance. This consensus definition of what level of resistance constitutes MDR was based on data showing that anti-TB chemotherapy was most likely to fail if the TB strain was resistant to isoniazid and rifampin at the beginning of treatment (*5*). The recognition that MDR strains further evolved with resistance to a selected group of reserve, or second-line, anti-TB drugs led to creation of the term XDR TB in 2006 (*6*). The definition of XDR TB is resistance to isoniazid and rifampin plus resistance to any fluoroquinolone and at least 1 of the 3 injectable second-line drugs used in TB treatment (amikacin, kanamicin, or capreomycin) (*7*).

### Malaria

Although the decreased sensitivity of malaria parasites to an antimalarial drug was first reported about a century ago in association with quinine, the term drug-resistant malaria was rarely used; resistance was not considered a major problem until the late 1950s, after chloroquine resistance emerged. Historically, chloroquine was widely used as the standard first-line drug against *P. falciparum*. Resistance was first detected on the Thailand–Cambodia and the Venezuela–Colombia borders, near areas where chloroquinated salt was used for malaria control, forcing the affected countries to begin switching to sulfadoxine–pyrimethamine (SP) in the 1970s. Resistance to SP developed quickly, again on the Thailand–Cambodia border. The spread of chloroquine and SP resistance to other parts of Asia and as far as Africa is well documented (*8*). A review of the development of drug-resistant malaria is available elsewhere (*9*). Only drug resistance in *P. falciparum* will be discussed in this article.

The term drug-resistant malaria originally referred to *P. falciparum* strains resistant to chloroquine, SP, or both. Multidrug resistance of *P. falciparum* is strictly defined as resistance to >2 antimalarial compounds of different chemical classes, recommended by the National Malaria Control Program (NMCP) (*10*). The Thailand–Cambodia border was the first area to be recognized as a multidrug resistant zone because of the successive failure of chloroquine, SP, and then mefloquine in the late 1980s. These antimalarial drugs belong to different chemical classes; all were designated as the first-line drugs against falciparum malaria by the Thai NMCP and were also used by the Cambodian NMCP.

## Relationship between Drug Resistance and Treatment

For new cases of TB, treatment is usually a 4-drug regimen of isoniazid, rifampin, pyrazinamide, and ethambutol for 2 months (intensive phase), followed by 4–6 months of only isoniazid and rifampin or isoniazid and ethambutol (continuation phase). The duration of this standardized regimen may vary, depending on a number of factors, including economic considerations and availability of culture-based diagnosis and monitoring. For example, United States guidelines, in contrast to those of most resource-constrained and developing countries, emphasize mycobacterial culture for TB diagnosis and recommend extending TB treatment for patients with cavities visible on chest radiograph and persistence of positive sputum cultures after 2 months of treatment.

To prevent inadequate drug ingestion and thereby resistance, staff in TB programs often directly observe patients ingesting their medications. Because directly observed therapy requires substantial human resources, the World Health Organization (WHO) recommends that directly observed therapy should be used any time that rifampin is administered. Many countries began using a combination of isoniazid and ethambutol, rather than isoniazid and rifampin, in the continuation phase, because of the cost of rifampin and an inability to provide directly observed therapy for the entire duration of this phase. The most common reason to vary the treatment regimen is documented drug resistance or a history of previous TB treatment, which is a risk factor for resistance development. In most developing countries, drug resistance data are sparse because confirmation of infection with *Mycobacterium tuberculosis* followed by drug susceptibility testing requires use of advanced molecular diagnostics and/or slower and more laborious culture methods. Therefore, patients are usually treated on the assumption that they are infected with a drug-susceptible strain. The drug regimen is usually changed only if the patient’s condition does not clinically improve, including having persistently positive sputum smears, after months of treatment. Retreatment protocols in most countries require prolonged therapy with essentially the same basic drugs before the patient is eligible to receive a drug regimen containing second-line drugs specifically for treatment of MDR TB.

Although multidrug regimens to prevent and treat drug-resistant TB were first evaluated in the 1950s, use of true combination therapy for malaria, the simultaneous use of >2 drugs (with independent modes of action and different chemical targets) to kill asexual blood-stage parasites, did not arise until much later. However, during the past decade, combination therapy has become the norm, intended to improve effectiveness and reduce the spread of resistance.

In uncomplicated malaria, an outpatient is usually treated with the first-line antimalarial drugs recommended by the local health authority for the malaria-endemic region in which the patient became infected. For example, a patient infected with *P. falciparum* on the eastern Thailand–Laos border would be treated with an artesunate–mefloquine combination plus primaquine at a government malaria clinic on the Thailand side of the border, or with an artemether–lumefantrine combination (Coartem; Novartis AG, Basel, Switzerland) on the Laos side of the border. For travelers returning to their home country outside a malaria-endemic area, different drugs may be prescribed. In none of these situations would a physician expect any laboratory tests to determine drug susceptibility before making a treatment decision. As an acute, potentially fatal disease, falciparum malaria requires effective treatment promptly.

For malaria, the geographic location in which infection is acquired is the primary determinant of the risk for a drug-resistant infection. Unlike MDR TB, the decision to treat and the treatment of MDR malaria do not require complex clinical and laboratory assessment of an individual patient’s isolate, except for severe malaria, which requires critical care capacity. For TB, the geographic area in which infection is acquired is not as reliable a determinant of the treatment choice. Despite wide differences in MDR TB prevalence across countries, the most reliable predictors of MDR risk for a TB patient are a history of prior treatment or known exposure to another case-patient (i.e., contact with an index MDR TB case-patient), not geography (*11*).

## Public Health Implications

Knowing the drug susceptibility pattern of a TB strain or whether a malaria infection was acquired in a specific malaria-endemic area helps not only with therapeutic decision making but also with predicting the patient’s prognosis. From the public health perspective, information on drug resistance is useful for strategic planning.

The proportion of TB case-patients infected with MDR strains, when stratified by previous treatment status, helps public health officials evaluate the intensity of community transmission and the strength of the TB program in curing patients. Unfortunately, the absence of continuous, systematic, representative, and timely drug susceptibility data, especially for second-line anti-TB drugs, is a major obstacle for the control of drug-resistant TB. Consequently, a large number of infectious MDR- and XDR TB cases globally may go undiagnosed.

For malaria, the level of drug resistance in a specific disease-endemic area is usually judged by in vivo therapeutic efficacy monitoring and in vitro drug susceptibility assays of malaria-infected blood specimens (*12*). To determine the trend of drug efficacy over years, each method requires sentinel sites, specially trained staff, and sustained efforts backed by steady public health policy. In fact, the simplest way to monitor the clinical efficacy of a given routine therapeutic regimen against the parasite is universal, comprehensive, posttherapeutic follow-up of the patients for 28–42 days. Such a procedure would draw early attention to the possibility of specific drug resistance, thus prompting appropriate investigations to avoid any possible delay in the confirmation of resistance; delay is an inherent shortcoming when observations are restricted to programmatic efficacy surveillance studies. Unfortunately, in practice this monitoring is sometimes difficult to achieve among some populations, such as mobile migrant populations, who are at high risk along many international borders.

Application of molecular surveys can also be useful for identifying regions at risk for emergence of antimalarial drug resistance and alerting program management of the need to conduct in-depth studies, thus allowing sufficient time for consideration of drug policy change (*8**,**13*). The availability of molecular markers for more drugs, markers with improved accuracy, improvement in the ease of assays, and the lowering of assay-associated costs will enhance the usefulness of molecular mapping of drug resistance.

For TB, the term XDR was created to describe not only TB strains that are resistant to more of the available drugs but also infections that are substantially more difficult to cure. For example, for patients co-infected with HIV, XDR TB is often fatal (*11*). An equivalent term for malaria does not yet exist, although infections with similar characteristics—resistance developed successively to more drugs and the lack of alternative drug choices—will represent identical challenges to control programs.

When the term MDR malaria was first introduced 2 decades ago, it was intended to describe resistance to new drug groups other than the common, standard antimalarial drugs used at that time, namely, chloroquine (a 4-aminoquinoline) and SP (antifolates). The term was first applied to the Thailand–Cambodia border after the emergence of mefloquine resistance. Artemisinin was introduced, in the form of artemisinin-based combination therapy (ACT), as a replacement for mefloquine monotherapy. Artesunate–mefloquine became the first ACT to be used for the control of MDR malaria. It was adopted as the first-line therapy for falciparum malaria by the Thai NMCP in 1995 and the Cambodian NMCP in 2000. The rapid antimalarial activity of artemisinin compounds means that they are most effective when used together with a partner drug that possesses a longer half-life (e.g., mefloquine), thus the rationale behind the combination. The effectiveness of artemisinin against MDR malaria is always cited as 1 of its advantageous characteristics (*14*). Recent evidence of the failure of artesunate–mefloquine combination therapy on the Thailand–Cambodia border and of resistance to artemisinin (*15**,**16*) has raised concerns about the failure of the last effective antimalarial drugs. The loss of artemisinins could negatively affect global public health because it would jeopardize effective malaria control, leading to increases in illness and eventually deaths. Malaria elimination, which has recently regained much interest, is also threatened by artemisinin failure (*17*).

## The Case for Defining XDR Malaria

The emergence of artemisinin resistance creates the need to define a new subgroup of drug-resistant malaria, XDR malaria**.** Such a label should be considered because it signifies the potential loss of artemisinin and highlights the threat of an expanded malaria-endemic area with poor ACT efficacy, similar to prior global spread of antimalarial drug resistance for other chemical classes. From the public health viewpoint, this situation must be handled carefully, beyond the existing WHO recommendations for malaria drug policy revision (*18*). The Cambodian and the Thai NMCPs are implementing special priority control measures against this high level of drug-resistant malaria. They are working together to contain artemisinin-resistant parasites; their ambitious goal is to eliminate falciparum malaria from this epicenter of resistance (*19*). To do so, they face a number of challenges, including the lack of a suitable alternative antimalarial drug for empiric treatment.

Not all malaria-endemic countries have well-documented, successive development of resistance to multiple drugs of different chemical classes, as has been documented on the Thailand–Cambodia border. Although artemisinin resistance has not been described outside Asia, a situation leading to similar development of extensively drug-resistant parasites will likely happen elsewhere. Africa is a primary concern because of its high prevalence of malaria. Chloroquine resistance and high-level SP resistance are highly prevalent in parts of Africa such as Kenya (*20*). A possible scenario of concern is the consolidation of amodiaquine and SP resistance and/or development of a new resistant strain against lumefantrine and/or mefloquine (which is known for its activity correlation with lumefantrine in vitro). In recent years, the combination of artemether and lumefantrine, or Coartem, was introduced in large scale to Africa. High rates of recurrence of *P. falciparum* infection have already been found in Zanzibar after Coartem therapy and include several cases of recrudescence associated with lumefantrine-resistant parasites (*21*). Although an ACT containing mefloquine has never been adopted by any African country, the artesunate–mefloquine combination is already common in some African markets. Antimalarial drugs that are not used, per national treatment guidelines, can be widely available enough, especially in the private sector of low-income countries, to induce selection pressure for resistance or cross-resistance.

After 2 decades of use, the term MDR malaria is not the trigger for action it once was. Designating a malaria-endemic area with artemisinin-resistant falciparum strains as an area with XDR malaria will signal an urgent need for action, such as ongoing public health attention and prioritizing funding and support. A similar sense of complacency with regard to MDR TB, the loss of treatment utility, and the need to instill urgency into global efforts to prevent and treat drug resistance led WHO to establish the term XDR TB in 2006 (*7*).

In most settings, the primary way to control XDR TB is to prevent its emergence through optimal treatment of drug-susceptible TB (*22*). Therefore, from a control program’s perspective, knowing that XDR TB exists in the community does not alter the general Stop TB strategy (www.who.int/tb/strategy/en/). However, specific determination of drug resistance is essential for clinical management. The cost of treating MDR or XDR TB cases that do emerge is high and requires extra public health resources, including a greatly expanded and upgraded laboratory network and access to specialized physician and nursing care. Similarly, the development of any drug resistance in malaria introduces an additional set of financial and operational challenges for a malaria control program.

For clinicians dealing with sporadic malaria cases outside a disease-endemic area, the term XDR malaria also deserves special attention. For example, Coartem is often prescribed for drug-resistant malaria*.* Given its potential limited efficacy against falciparum malaria in western Cambodia (*23*) and activity correlation between mefloquine and lumefantrine in vitro, its prescription for patients from XDR malaria areas will need to be reconsidered.

Introducing the term XDR malaria in association with artemisinin resistance should not discourage the deployment of ACTs in Africa, where it is hoped that these regimens will contribute to substantial reduction of malaria incidence and deaths. However, it will help alert countries that have recently adopted ACTs that resistance is possible and that vigilance in monitoring for resistance and reinforcement of rational drug use are essential. Control programs sometimes strongly strive for universal access to ACTs but are unable to regulate or ensure their rational use, especially when ACTs are widely available in the private and informal healthcare sectors (*24*). Such a balance of concerns should be redressed.

## Conclusions

For TB and malaria control, providing specific labels to drug-resistant strains benefits individual patient care and public health. For TB, agreement on specific definitions for MDR and XDR TB facilitated epidemiologic assessment, program planning, laboratory capacity enhancement, and development of standardized treatment regimens. For malaria, the rapid increase in the prevalence of drug-resistant malaria globally and emerging artemisinin resistance in Southeast Asia show that the time to define and combat XDR malaria has arrived.

## References

[1] World Health Organization. Global tuberculosis control– epidemiology, strategy, financing. WHO report 2009. WHO/HTM/TB/2009.411. Geneva: The Organization; 2009 [cited 2010 Apr 30]. http://www.who.int/tb/publications/global_report/2009/en/index.html

[2] World Health Organization. World malaria report 2009. Geneva: The Organization, 2009 [cited 2010 Apr 30]. http://www.who.int/malaria/world_malaria_report_2009/en/index.html

[3] Crofton J, Mitchison D. Streptomycin resistance in pulmonary tuberculosis. BMJ. 1948;2:1009–15. 10.1136/bmj.2.4588.100918100441PMC2092236

[4] Mitchinson DA. The diagnosis and therapy of tuberculosis during the past 100 years. Am J Respir Crit Care Med. 2005;171:699–706. 10.1164/rccm.200411-1603OE15709048

[5] Mitchison DA, Nunn AJ. Influence of initial drug resistance on the response to short-course chemotherapy of pulmonary tuberculosis. Am Rev Respir Dis. 1986;133:423–30.242024210.1164/arrd.1986.133.3.423

[6] Centers for Diseases Control and Prevention. Emergence of *Mycobacterium tuberculosis* with extensive resistance to second-line drugs—worldwide, 2000–2004. MMWR. 2006;55:301–5.16557213

[7] World Health Organization. Report of the meeting of the WHO Global Task Force on XDR-TB; 2006 Oct 9–10; Geneva, Switzerland. Geneva: The Organization; 2007 [cited 2010 Apr 30]. http://whqlibdoc.who.int/hq/2007/WHO_HTM_TB_2007.375_eng.pdf

[8] Plowe CV. The evolution of drug-resistant malaria. Trans R Soc Trop Med Hyg. 2009;103(Suppl 1):S11–4. 10.1016/j.trstmh.2008.11.00219084883PMC2723787

[9] Talisuna AO, Bloland P, D'Alessandro U. History, dynamics, and public health importance of malaria parasite resistance. Clin Microbiol Rev. 2004;17:235–54. 10.1128/CMR.17.1.235-254.200414726463PMC321461

[10] Wernsdorfer WH. Epidemiology of drug resistance in malaria. Acta Trop. 1994;56:143–56. 10.1016/0001-706X(94)90060-48203301

[11] Granich RM, Oh P, Lewis B, Porco TC, Flood J. Multidrug resistance among persons with tuberculosis in California, 1994–2003. JAMA. 2005;293:2732–9. 10.1001/jama.293.22.273215941802

[12] World Health Organization. Susceptibility of *Plasmodium falciparum* to antimalarial drugs. Report on global monitoring, 1996–2004. WHO/HTM/MAL/2005.1103; Geneva: The Organization; 2005 [cited 2010 Apr 30]. http://www.who.int/malaria/publications/atoz/whohtmmal20051103/en/index.html

[13] Beck HP. How does molecular epidemiology help to understand malaria? Trop Med Int Health. 1999;4:1–3. 10.1046/j.1365-3156.1999.00353.x10203166

[14] World Health Organization. Malaria: guidelines for the treatments of malaria, second edition. WHO/HTM/MAL/2006.1108. Geneva: The Organization; 2006 [cited 2010 Apr 30]. http://www.who.int/malaria/publications/atoz/9789241547925/en/index.html

[15] Wongsrichanalai C, Meshnick SR. Declining artesunate–mefloquine efficacy against falciparum malaria on the Cambodia–Thailand border. Emerg Infect Dis. 2008;14:716–9. 10.3201/eid1405.07160118439351PMC2600243

[16] Dondorp AM, Nosten F, Yi P, Das D, Phyo AP, Tarning J, Artemisinin resistance in *Plasmodium falciparum* malaria. N Engl J Med. 2009;361:455–67. Medline 10.1056/NEJMoa0808859PMC349523219641202

[17] White NJ. The role of anti-malarial drugs in eliminating malaria. Malar J. 2008;7(Suppl 1):S8. 10.1186/1475-2875-7-S1-S819091042PMC2604872

[18] World Health Organization. Global malaria control and elimination: report of a meeting on containment of artemisinin tolerance, 19 January 2008, Geneva, Switzerland. Geneva: WHO; 2008 [cited 2010 Apr 30]. http://www.who.int/malaria/publications/atoz/9789241596817/en/index.html

[19] World Health Organizaion. Pacific Region. Minutes of an informal consultation on resource mobilization of the containment of artemisinin-tolerant malaria on the Cambodia–Thailand border, Phnom Penh, Cambodia; 2008 Jun 17–18. WHO/WPRO report series no. RS/2008/GE/28(CAM). Manila (the Phillipines): The Organization; 2008 [cited 2010 Apr 30]. http://www.whothailand.org/LinkFiles/Mekong_Malaria_Programme_MMP_Informal_Consultation_on_Resource_Mobilization_for_the_Containment_of_Artemisinin_JUN08.pdf

[20] Zhong D, Afrane Y, Githeko A, Cui L, Menge DM, Yan G. Molecular epidemiology of drug-resistant malaria in western Kenya highlands. BMC Infect Dis. 2008;8:105. 10.1186/1471-2334-8-10518671871PMC2533336

[21] Sisowath C, Strömberg J, Mårtensson A, Msellem M, Obondo C, Björkman A, In vivo selection of *Plasmodium falciparum pfmdr1* 86N coding alleles by artemether–lumefantrine (Coartem). J Infect Dis. 2005;191:1014–7. 10.1086/42799715717281

[22] Dye C. Doomsday postponed? Preventing and reversing epidemics of drug-resistant tuberculosis. Nat Rev Microbiol. 2009;7:81–7. 10.1038/nrmicro204819079354

[23] Denis MB, Tsuyuoka R, Lim P, Lindegardh N, Yi P, Top SN, Efficacy of artemether–lumefantrine for the treatment of uncomplicated falciparum malaria in northwest Cambodia. Trop Med Int Health. 2006;11:1800–7. 10.1111/j.1365-3156.2006.01739.x17176344

[24] Yeung S, Van Damme W, Socheat D, White NJ, Mills A. Access to artemisinin combination therapy for malaria in remote areas of Cambodia. Malar J. 2008;7:96. 10.1186/1475-2875-7-9618510724PMC2430580

# Review article: Extensive Drug Resistance in Malaria and Tuberculosis

**DOI:** 10.3201/eid1607.091840

**Authors:** Chansuda Wongsrichanalai, Jay K. Varma, Jonathan J. Juliano, Michael E. Kimerling, John R. MacArthur

**Affiliations:** Author affiliations: US Agency for International Development, Bangkok, Thailand (C. Wongsrichanalai, J.R. MacArthur);; US Centers for Disease Control and Prevention, Beijing, People’s Republic of China (J.K. Varma);; US Centers for Disease Control and Prevention, Atlanta, Georgia, USA (J.K. Varma, J.R. MacArthur);; University of North Carolina School of Medicine, Chapel Hill, North Carolina, USA (J.J. Juliano);; The Bill and Melinda Gates Foundation, Seattle, Washington, USA (M.E. Kimerling)

## Earning CME Credit

To obtain credit, you should first read the journal article. After reading the article, you should be able to answer the following, related, multiple-choice questions. To complete the questions and earn continuing medical education (CME) credit, please go to http://cme.medscape.com/viewpublication/30063. Credit cannot be obtained for tests completed on paper, although you may use the worksheet below to keep a record of your answers. You must be a registered user on Medscape.com. If you are not registered on Medscape.com, please click on the New Users: Free Registration link on the left hand side of the website to register. Only one answer is correct for each question. Once you successfully answer all post-test questions you will be able to view and/or print your certificate. For questions regarding the content of this activity, contact the accredited provider, CME@medscape.net. For technical assistance, contact CME@webmd.net. American Medical Association’s Physician’s Recognition Award (AMA PRA) credits are accepted in the US as evidence of participation in CME activities. For further information on this award, please refer to http://www.ama-assn.org/ama/pub/category/2922.html. The AMA has determined that physicians not licensed in the US who participate in this CME activity are eligible for *AMA PRA Category 1 Credits*™. Through agreements that the AMA has made with agencies in some countries, AMA PRA credit is acceptable as evidence of participation in CME activities. If you are not licensed in the US and want to obtain an AMA PRA CME credit, please complete the questions online, print the certificate and present it to your national medical association.

## Article Title: Extensive Drug Resistance in Malaria and Tuberculosis

## CME Questions

**1. You are seeing a couple who recently emigrated from Uganda. One of these patients, a 32-year-old woman, has a history of active tuberculosis (TB) and said she was treated with several drugs for 2 months, but she was told they were not working.**

**According to international consensus, multidrug-resistant (MDR) TB is defined by resistance to at least which of the following medications?**

A. Rifampin only

B. Rifampin and isoniazid

C. Isoniazid and ethambutol

D. Ethambutol, pyrazinamide, and Rifampin

**2. Which of the following statements regarding the treatment of this patient with TB is most accurate?**

A. Guidelines in the United States now recommend extending 4-drug treatment for TB based on the patients' symptoms alone

B. The World Health Organization recommends directly observed therapy (DOT) for treatment with Rifampin

C. Previous TB treatment does not affect her risk of harboring resistant organisms

D. TB treatment should be changed when there is no clinical improvement after 2 weeks

**3. The other patient is a 31-year-old man with intermittent fever and headache for 3 weeks. He is hospitalized, and malaria is diagnosed.**

**Which of the following general principles regarding the treatment of this patient is most accurate?**

A. Multidrug resistance in *Plasmodium falciparum* is defined by resistance to more than 2 operational antimalarial compounds from 2 different classes

B. The use of combination antimalarial therapy has not improved efficacy or reduced resistance

C. The decision to treat MDR malaria is based on complicated laboratory data

D. Geography has little impact on the treatment choice of malaria

**4. Which of the following statements regarding drug-resistant malaria is most accurate?**

A. The simplest way to monitor the efficacy of a given therapeutic regimen is through molecular characterization

B. There is no known resistance to the combination of artesunate and mefloquine

C. Artemisinin resistance is now common in Africa

D. Rates of artemisinin-based combination resistance need to be monitored in Africa

## Activity Evaluation

**Table:** 

1. The activity supported the learning objectives.				
Strongly Disagree				Strongly Agree
1	2	3	4	5
2. The material was organized clearly for learning to occur.				
Strongly Disagree				Strongly Agree
1	2	3	4	5
3. The content learned from this activity will impact my practice.				
Strongly Disagree				Strongly Agree
1	2	3	4	5
4. The activity was presented objectively and free of commercial bias.				
Strongly Disagree				Strongly Agree
1	2	3	4	5
